# Net clinical benefit of warfarin in individuals with atrial fibrillation across stroke risk and across primary and secondary care

**DOI:** 10.1136/heartjnl-2016-309910

**Published:** 2016-08-31

**Authors:** Victoria Allan, Amitava Banerjee, Anoop Dinesh Shah, Riyaz Patel, Spiros Denaxas, Juan-Pablo Casas, Harry Hemingway

**Affiliations:** Farr Institute of Health Informatics Research, Institute of Health Informatics, University College London, London, UK

**Keywords:** Stroke

## Abstract

**Objective:**

To investigate net clinical benefit (NCB) of warfarin in individuals with atrial fibrillation (AF) across stroke risk and across primary and secondary care.

**Methods:**

We conducted a linked electronic health record cohort study of 70 206 individuals with initial record of diagnosis of AF in primary (n=29 568) or secondary care (n=40 638) in England (1998–2010). We defined stroke risk according to the CHA_2_DS_2_-VASc score, and followed individuals over a median 2.2 years for 7005 ischaemic strokes (IS) and for 906 haemorrhagic strokes (HS). We calculated incidence rates (IRs) and 95% CIs per 100 person-years (PYs) (IR (95% CI)/100 PY) of IS and HS, with and without use of warfarin, and the NCB (ie, number of IS avoided) per 100 PYs of warfarin use (NCB (95% CI)/100 PY).

**Results:**

Compared with individuals with initial record of diagnosis in secondary care, those in primary care had lower scores of IS risk (CHA_2_DS_2_-VASc≤2: 30.8% vs 20.6%), and lower overall incidence of IS (IR (95% CI)/100 PY: 2.3 (2.2 to 2.4) vs 4.3 (4.2 to 4.4), p value=0.00); however among individuals with CHA_2_DS_2_-VASc=0, 1 or 2 there were no differences in IS rate between those with initial record of diagnosis in primary care or secondary care (IR (95% CI)/100 PY: 0.2 (0.1 to 0.3) vs 0.3 (0.2 to 0.5), p value=0.16), (IR (95% CI)/100 PY: 0.6 (0.4 to 0.7) vs 0.7 (0.6 to 0.9), p value=0.08) and (IR (95% CI)/100 PY: 1.1 (1.00 to 1.3) vs 1.4 (1.2 to 1.6), p value=0.05), respectively. For CHA_2_DS_2_-VASc=0, 1 and 2, IRs of IS with versus without warfarin were (IR (95% CI)/100 PY: 0.4 (0.2 to 0.8) vs 0.2 (0.1 to 0.3), p value=0.16), (IR (95% CI)/100 PY: 0.4 (0.3 to 0.7) vs 0.7 (0.6 to 0.8), p value=0.03) and (IR (95% CI)/100 PY: 0.8 (0.7 to 1.0) vs 1.4 (1.3 to 1.6), p value=0.00), respectively. We found a significant positive NCB of warfarin from CHA_2_DS_2_-VASc≥2 in men (NCB (95% CI)/100 PY: 0.5 (0.1 to 0.9)) and from CHA_2_DS_2_-VASc≥3 in women (NCB (95% CI)/100 PY: 1.5 (1.1 to 1.9)).

**Conclusions:**

CHA_2_DS_2_-VASc accurately stratifies IS risk in individuals with AF across both primary and secondary care. However, the incidence rate of ischaemic stroke at CHA_2_DS_2_-VASc=1 are lower than previously reported, which may change the decision to start anticoagulation with warfarin in these individuals.

## Introduction

CHA_2_DS_2_-VASc (congestive heart failure, hypertension, age ≥75 years, diabetes mellitus, history of stroke or thromboembolism, vascular disease, age 65–74 years and female sex) is the most widely used and validated clinical prediction score for assessment of ischaemic stroke (IS) risk in individuals with atrial fibrillation (AF). The score ranges from 0 to 9, and assigns 1 or 2 points for each stroke risk factor.^1^ CHA_2_DS_2_-VASc aims to identify individuals with the lowest stroke risk (CHA_2_DS_2_-VASc=0) in whom prevention with anticoagulants is not advised. The advice for individuals with one stroke risk factor (CHA_2_DS_2_-VASc=1) varies in current clinical practice guidelines, as it remains uncertain whether the benefits of anticoagulants outweigh the harms.^2^ ^3^ ^4^

**Figure 1:**
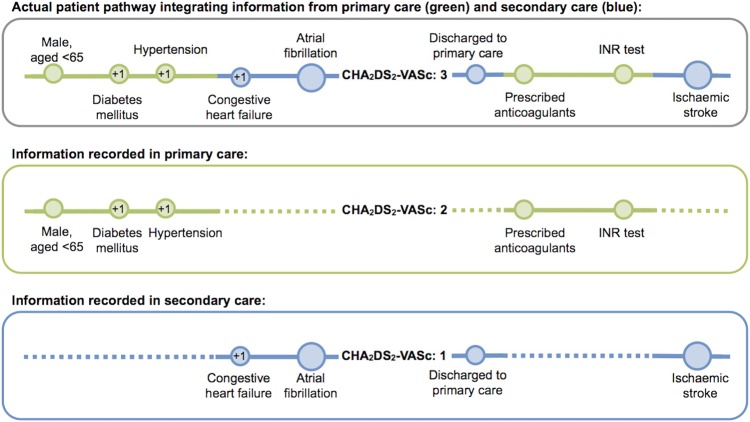
Example of one patient's actual pathway and how information is captured in primary care and secondary care records. This illustrates how lack of integration of primary and secondary care information may lead to underestimation of CHA_2_DS_2_-VASc scores. Patient interactions occurring in primary and secondary care are colour-coded green and blue, respectively. +1 indicates scoring of one CHA_2_DS_2_-VASc risk factor point. As shown, this patient has CHA2DS2-VASc=1 based on secondary care records only, CHA2DS2-VASc=2 based on primary care records only, but is truly CHA2DS2-VASc=3 based on integrated primary and secondary care records. INR, International Normalised Ratio.

A recent systematic review of incidence rates (IRs) of IS in individuals with CHA_2_DS_2_-VASc=1 reported highly heterogeneous annualised rates ranging from 0.1% to 6.6% across studies, with wide uncertainty (0–3.23%) in the pooled estimate of 1.6%.^5^ Among the 10 included studies, there were 0 studies that involved individuals across both primary and secondary care. Primary care accounts for over 40% of initial AF diagnoses.^6^ Therefore without the inclusion of these individuals, it is unclear whether previously reported stroke rates are representative of the full patient pathway. Furthermore, in many countries (including the UK, Denmark and Sweden) clinical information coded and recorded electronically in secondary care is not integrated with that of primary care.^7^ Thus, without the inclusion of risk factors diagnosed in primary care, previous studies based on secondary care data may have inaccurately calculated individuals' CHA_2_DS_2_-VASc scores (figure 1).^8^ Lastly, without conducting net clinical benefit (NCB) analyses, which weigh up benefits and harms of anticoagulants, the review was limited in the extent to which it could inform recommendations for clinical practice guidelines.

We therefore implemented the CHA_2_DS_2_-VASc score in a large, nationally representative linked electronic health record (EHR) study of 70 206 individuals with initial record of diagnosis of non–valvular AF in primary or secondary care in England in 1998–2010. We selected an observation period prior to the introduction of direct oral anticoagulants (DOACs), in order to more accurately study outcomes with warfarin. Our objectives were (1) to investigate incidence of IS in individuals with AF across stroke risk and across primary and secondary care, and (2) to consider benefits and harms of warfarin in NCB analyses.

## Methods

### Data sources

We used available data from the population-based CALIBER (ClinicAl research using LInked Bespoke studies and EHRs) study.^7^ CALIBER connects four national sources of EHRs in England, including a subset of primary care data from the Clinical Practice Research Datalink GP OnLine Database (CPRD GOLD),^9^ secondary care data from Hospital Episode Statistics (HES),^10^ data on admissions to hospital with an acute coronary syndrome from the Myocardial Ischaemic National Audit Project^11^ and cause-specific mortality data from the Office of National Statistics (ONS).^12^ The denominator population comprises individuals captured in CPRD GOLD, who are representative of the UK population in terms of gender, age, ethnicity^13^ and overall mortality.^14^ For this analysis we used CPRD GOLD, HES and ONS. CPRD GOLD is coded using Read clinical terms,^15^ and HES and ONS using the International Classification of Diseases V.10 (ICD–10).^16^ A key objective of CALIBER is to facilitate transparent and reproducible research of these data through the publication of disease phenotypes and tools to support statistical analysis, and these can be found freely available at caliberresearch.org. CALIBER research has been shown to replicate known and discover new associations with risk factors for the onset of disease.^17–20^

### Selection of individuals with non-valvular AF

We used the previously reported CALIBER phenotype algorithm for AF to identify all individuals with a diagnosis in primary or secondary care between 1998 and 2010.^6^ The algorithm includes primary and secondary care (main and subsidiary) recorded diagnoses, and inferred diagnoses based on warfarin prescriptions without prior thromboembolic disease. In order to define non-valvular AF we then excluded individuals with a record of mitral valve disease, rheumatic mitral regurgitation, and prosthetic mitral, aortic or unspecified valve replacements,^21^ based on 45 Read codes, 9 ICD–10 codes and 11 operation and procedure codes (see online supplementary table S1 for the code list).

10.1136/heartjnl-2016-309910.supp1 supplementary data

### CHA_2_DS_2_-VASc

We generated baseline CHA_2_DS_2_-VASc scores for each individual by assigning 1 or 2 points for each stroke risk factor. Risk factors were defined according to existing CALIBER phenotypes (available at caliberresearch.org), which use all available clinical information across the linked primary and secondary care records. In brief, age and sex were obtained from general practice registration information; congestive heart failure, diabetes mellitus (types I, II and unclassified), history of stroke or thromboembolism (stroke, transient ischaemic attack or systemic embolism), and vascular disease (myocardial infarction (MI) or peripheral artery disease (PAD)) from clinical diagnosis codes; and hypertension from diagnosis codes, two or more values of systolic or diastolic blood pressure measurements above UK diagnostic thresholds of 140/90 mm Hg,^22^ or through repeat prescriptions for blood pressure lowering medications.

### Warfarin

We considered warfarin use throughout the entire study period and extracted information on prescriptions and international normalised ratio (INR) tests from primary care records. Prescriptions data include drug type and date of administration but do not have information on pharmacy collections. Individuals were considered to be using warfarin continuously during follow-up if a prescription or INR test was administered every 30 days. This allowed each individual's follow-up time to be divided into periods with and without use of warfarin.^23^

### Follow-up and end points

Individuals entered the study at their earliest coded diagnosis of AF during January 1998 and March 2010, provided they were aged 18 years and over and with a minimum of 1 year of continuous registration at a general practice with acceptable data recording standards. We followed individuals for clinical diagnoses of IS and unclassified strokes, and for haemorrhagic strokes (HSs) (intracerebral and subarachnoid haemorrhage) as recorded in primary or secondary care and mortality registry records. ISs and unclassified strokes were combined, as it has previously been shown that 87% of all strokes are ischaemic.^24^ A clearance period of 2 weeks was imposed from date of recorded AF diagnosis, such that any stroke occurring during this time was attributed to baseline risk and not counted as an end point. The rationale is that AF is commonly first detected when an individual presents with a complication, such as stroke.^25^ Clearance periods are also imperative when analysing linked EHRs to avoid double counting, as the same event can be recorded more than once, and in multiple data sources.^26^ Total follow-up time for each individual was calculated as the number of days from the end of the clearance period to the point of censoring. Individuals were censored in the event of an IS or HS end point, death (from a cause other than a stroke end point), transfer out of general practice, or last date of data collection.

### Data analysis

We compared baseline CHA_2_DS_2_-VASc risk factors in individuals with initial record of diagnosis in primary and secondary care using the Student's t-test or χ^2^ test, and these are presented as proportions, means (SD) and medians (range, IQR), as appropriate. We assessed the completeness of recording CHA_2_DS_2_-VASc risk factors in each data source using absolute proportions, that is, number (%) of total cases captured in primary care records, and in secondary care records, compared with both data sources linked. We calculated IRs and 95% CIs per 100 person-years (PYs) (IR (95% CI)/100 PY) for IS and HS by dividing the number of end points by the accrued number of PYs. We assessed whether IRs were robust by comparing with estimates adjusted for propensity score quintiles. This accounts for whether individuals were more likely or less likely to receive treatment with warfarin. We conducted NCB analyses comparing number of ISs avoided, against number of HSs experienced per 100 PYs of warfarin use (NCB (95% CI)/100 PY). We used the formula: (IS rate_without warfarin_−IS rate_with warfarin_)−1.5 (HS rate_with warfarin_−HS rate_without warfarin_), whereby a positive estimate indicates a treatment benefit, and a negative estimate indicates treatment harm.^27^ We regarded the NCB as significant if the 95% CI did not span both the positive and negative scales. All analyses were conducted in Stata/SE V.13 and figures were generated in R (V.3.2.0).

## Results

### Population characteristics

#### Population overall

The overall study population comprised 70 206 individuals with non-valvular AF with median age of 77.9 years (range: 18.0–108.7, IQR: 15.1), and median follow-up of 2.20 years (range: 0.03–12.2, IQR: 4.2). Of the individuals 34 286 (48.8%) were women, and 2486 (3.5%) had CHA_2_DS_2_-VASc=0, 5637 (8.0%) had CHA_2_DS_2_-VASc=1 and 9339 (13.3%) had CHA_2_DS_2_-VASc=2. The mean (SD) CHA_2_DS_2_-VASc score of the overall population was 3.7 (1.8).

#### Individuals with initial record of diagnosis in primary versus secondary care

Of the individuals 29 568 (42.1%) had initial record of diagnosis of AF in primary care, and 40 638 (57.9%) had initial record of diagnosis in secondary care. Individuals with initial record of diagnosis in secondary care were older (median (IQR) age: 79.1 (15.0) years vs 76.5 (14.8) years), more likely to be female (50.1% vs 47.1%) and were less likely to have CHA_2_DS_2_-VASc=0 (2.9% vs 4.4%), CHA_2_DS_2_-VASc=1 (6.6% vs 10.1%) or CHA_2_DS_2_-VASc=2 (11.1% vs 6.6%) than those with initial record of diagnosis in primary care. The mean (SD) CHA_2_DS_2_-VASc score among individuals with initial record of diagnosis of AF in primary care compared with secondary care was 3.3 (1.7) vs 4.0 (1.8). As table 1 shows, individuals with initial record of diagnosis in secondary care had a higher proportion of all CHA_2_DS_2_-VASc risk factors.

**Table 1:** Comparison of baseline CHA_2_DS_2_-VASc risk factors in individuals with initial record of diagnosis in primary or secondary care

	Initial record of diagnosis					
	Primary care		Secondary care		Population overall	
Number of individuals	29 568		40 638		70 206	
	N	Per cent	N	Per cent	N	Per cent
**C**ongestive heart failure	4768	16.1	12 664	31.2	17 432	24.8
**H**ypertension	23 946	81.0	33 817	83.2	57 763	82.3
Diagnosis	16 616	56.2	25 273	62.2	41 889	59.7
Blood pressure medication	20 979	71.0	30 164	74.2	51 143	72.9
Blood pressure measures	17 281	58.4	22 329	55.0	39 610	56.4
**A**ge ≥ 75 years^2^	16 318	55.2	25 872	63.7	42 190	60.1
**D**iabetes	3316	11.2	6673	16.4	9989	14.2
**S**troke/transient ischaemic attack/ systemic embolism^2^	3887	13.2	8938	22.0	12 825	18.3
**V**ascular disease	4049	13.7	9783	24.1	13 832	19.7
Myocardial infarction	2635	8.9	6950	17.1	9585	13.7
Peripheral vascular disease	1776	6.0	3927	9.7	5703	8.1
**A**ge 65–74 years	7744	26.2	8552	21.0	16 296	23.2
**S**ex **c**ategory (female)	13 930	47.1	20 356	50.1	34 286	48.8
CHA_2_DS_2_-VASc scores						
0	1305	4.4	1181	2.9	2486	3.5
1	2972	10.1	2665	6.6	5637	8.0
2	4820	16.3	4519	11.1	9339	13.3
3	6663	22.5	7107	17.5	13 770	19.6
4	7332	24.8	9578	23.6	16 910	24.1
5	3712	12.6	7514	18.5	11 226	16.0
6	1866	6.3	4906	12.1	6772	9.7
7	724	2.5	2341	5.8	3065	4.4
8	156	0.5	707	1.7	863	1.2
9	18	0.1	120	0.3	138	0.2

#### Individuals with versus without use of warfarin

Of the individuals 30 067 (42.8%) underwent at least one period of warfarin use during follow-up; 50.1% of these had initial record of diagnosis in primary care (n=15 077) and 49.9% had initial record of diagnosis in secondary care (n=14 990). Individuals without use of warfarin were older (median (IQR) age: 80.7 (15.2) years vs 74.9 (13.4) years) and had a higher proportion of heart failure, but there was no difference in diagnosed hypertension, vascular disease (MI or PAD), diabetes and previous strokes, when compared with those with at least one period of warfarin use (see online supplementary table S2).

#### Men versus women

Women were older (median (IQR) age: 80.8 (13.3) years vs 74.9 (15.9) years) and had a higher proportion of heart failure, hypertension and previous strokes, while vascular disease (MI and PAD) and diabetes were more common in men (see online supplementary table S3).

### Completeness of risk factors and reclassification of CHA_2_DS_2_-VASc scores

The completeness of recording CHA_2_DS_2_-VASc risk factors in primary and secondary care records ranged from 40.4% to 73.7% complete in secondary care records, and from 69.1% to 99.0% in primary care records (see online supplementary table S4 and figure S1). Among individuals with initial record of diagnosis in secondary care, 975 (45.2%) were reclassified from CHA_2_DS_2_-VASc=0 to CHA_2_DS_2_-VASc≥1 and 2172 (53.7%) from CHA_2_DS_2_-VASc=1 to CHA_2_DS_2_-VASc≥2 when scores were calculated using linked primary–secondary care records. Only 15 (1.1%) individuals with initial record of diagnosis in primary care were reclassified from CHA_2_DS_2_-VASc=0 to CHA_2_DS_2_-VASc≥1 and 81 (2.7%) from CHA_2_DS_2_-VASc=1 to CHA_2_DS_2_-VASc≥2. For CHA_2_DS_2_-VASc scores calculated based on primary and secondary care records compared with both data sources linked, see online supplementary table S5.

### Stroke incidence

#### IS incidence in individuals with initial record of diagnosis in primary or secondary care

**Figure 2:**
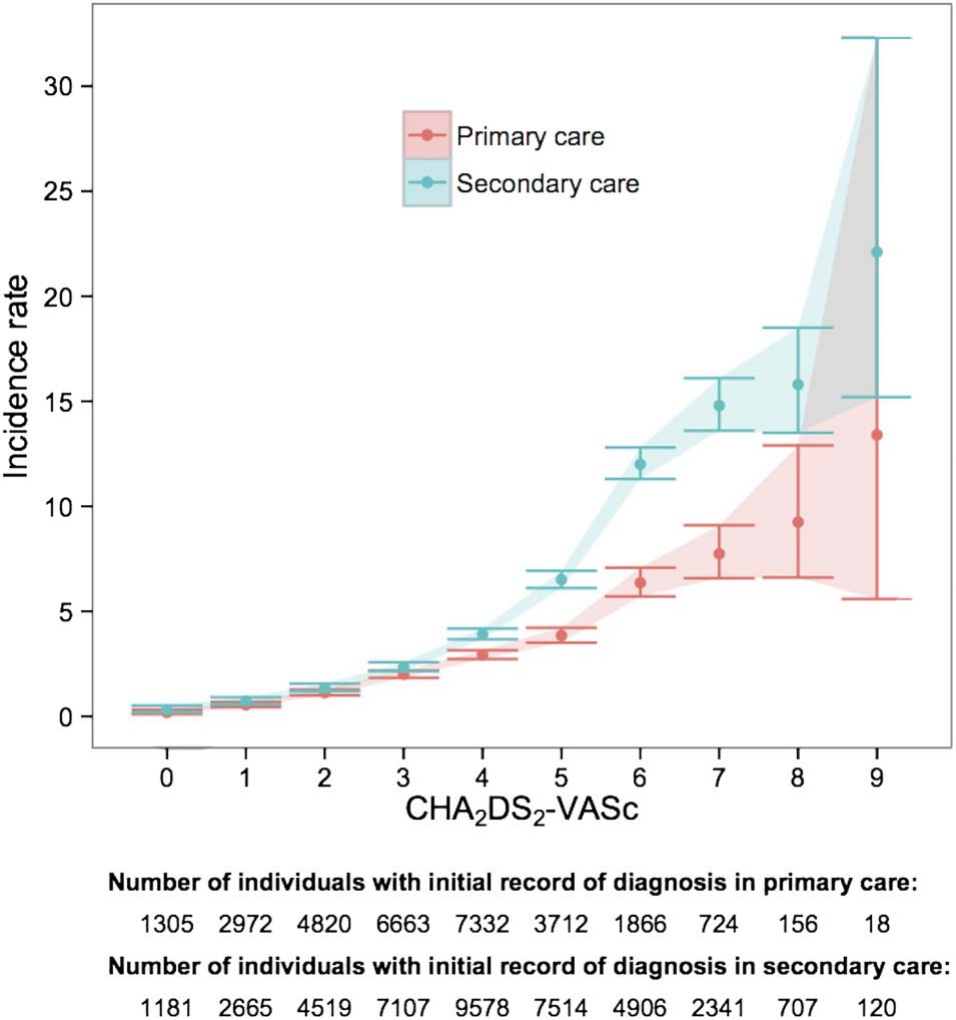
Incidence rates (95% CIs) per 100 person-years of ischaemic stroke by CHA_2_DS_2_-VASC scores in individuals with initial record of diagnosis in primary or secondary care.

Seven thousand and five ISs occurred over 216 446 PYs, with IR (95% CI)/100 PY of 3.2 (3.2 to 3.3). Compared with individuals with initial record of diagnosis in secondary care, those in primary care had lower overall IS incidence (IR (95% CI)/100 PY: 2.3 (2.2 to 2.4) vs 4.3 (4.2 to 4.4), p value=0.00), however as figure 2 shows there were no differences in incidence at CHA_2_DS_2_-VASc=0 (IR (95% CI)/100 PY: 0.2 (0.1 to 0.3) vs 0.3 (0.2 to 0.5), p value=0.16), CHA_2_DS_2_-VASc=1 (IR (95% CI)/100 PY: 0.6 (0.4 to 0.7) vs 0.7 (0.6 to 0.9), p value=0.08) or CHA_2_DS_2_-VASc=2 (IR (95% CI)/100 PY: 1.1 (1.00 to 1.3) vs 1.4 (1.2 to 1.6), p value=0.05). IRs in individuals with initial record of diagnosis in primary or in secondary care across all CHA_2_DS_2_-VASc scores are provided in table 2.

**Table 2:** Incidence rates (95% CIs) per 100 person-years of ischaemic stroke by CHA_2_DS_2_-VASC scores in individuals with initial record of diagnosis in primary or secondary care

	Initial record of diagnosis						
	Primary care		Secondary care		p Value	Population overall	
	Events	Rate	Events	Rate		Events	Rate
CHA_2_DS_2_-VASc scores							
0	12	0.2 (0.1 to 0.3)	16	0.3 (0.2 to 0.5)	0.16	28	0.2 (0.2 to 0.4)
1	77	0.6 (0.4 to 0.7)	76	0.7 (0.6 to 0.9)	0.08	153	0.6 (0.5 to 0.7)
2	244	1.1 (1.0 to 1.3)	209	1.4 (1.2 to 1.6)	0.05	453	1.2 (1.1 to 1.3)
3	528	2.0 (1.8 to 2.2)	485	2.4 (2.2 to 2.6)	0.01	1013	2.2 (2.0 to 2.3)
4	766	2.9 (2.7 to 3.2)	907	3.9 (3.7 to 4.2)	0.00	1673	3.4 (3.2 to 3.6)
5	450	3.9 (3.5 to 4.2)	966	6.5 (6.1 to 6.9)	0.00	1416	5.3 (5.1 to 5.6)
6	332	6.4 (5.7 to 7.1)	1028	12.0 (11.3 to 12.8)	0.00	1360	9.9 (9.4 to 10.4)
7	146	7.7 (6.6 to 9.1)	546	14.8 (13.6 to 16.1)	0.00	692	12.4 (11.5 to 13.3)
8	34	9.3 (6.6 to 12.9)	151	15.8 (13.5 to 18.5)	0.00	185	13.9 (12.1 to 16.1)
9	5	13.4 (5.6 to 32.3)	27	22.1 (15.2 to 32.3)	0.31	32	20.0 (14.2 to 28.4)
**0–9**	**2594**	**2.3 (2.2** to **2.4)**	**4411**	**4.3 (4.2** to **4.42)**	0.00	**7005**	**3.2 (3.2** to **3.3)**

Bold text is used to differentiate the rates among individual scores 0,1, 2, …, 9, from all scores 0 to 9 combined.

#### IS incidence by warfarin use

**Figure 3:**
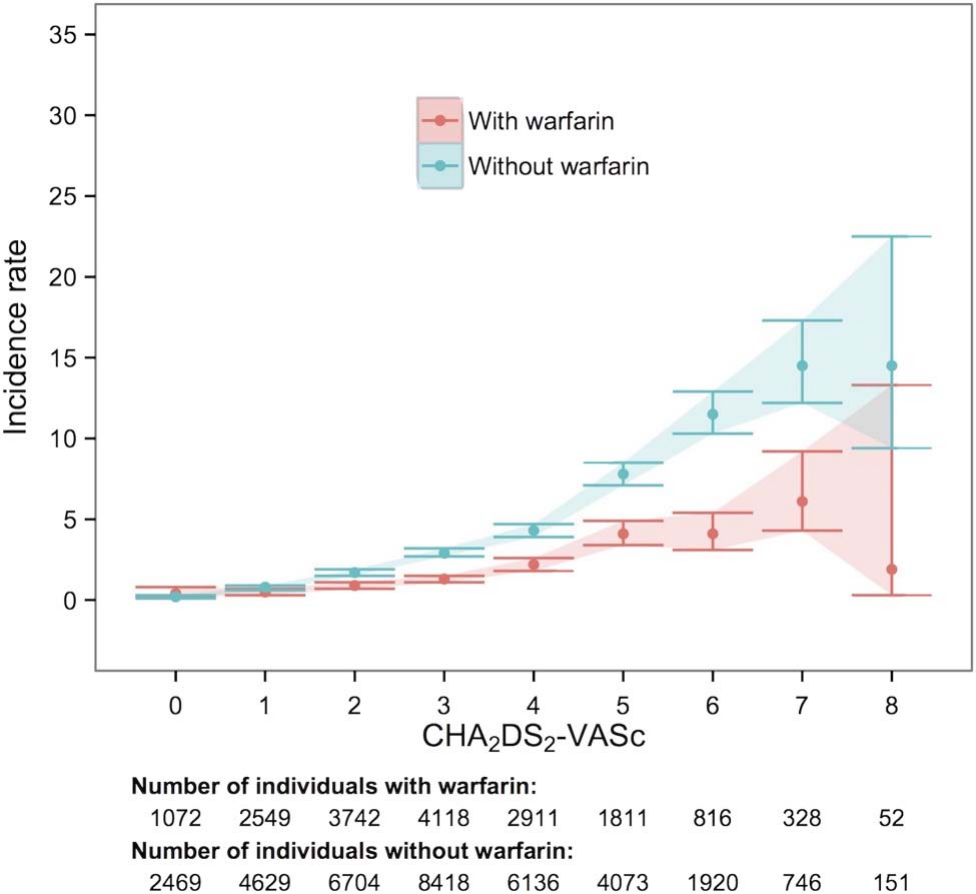
Incidence rates (95% CIs) per 100 person-years of ischaemic stroke in men by CHA_2_DS_2_-VASC scores, and use of warfarin. Individuals could contribute follow-up time to periods with and without warfarin.

**Figure 4:**
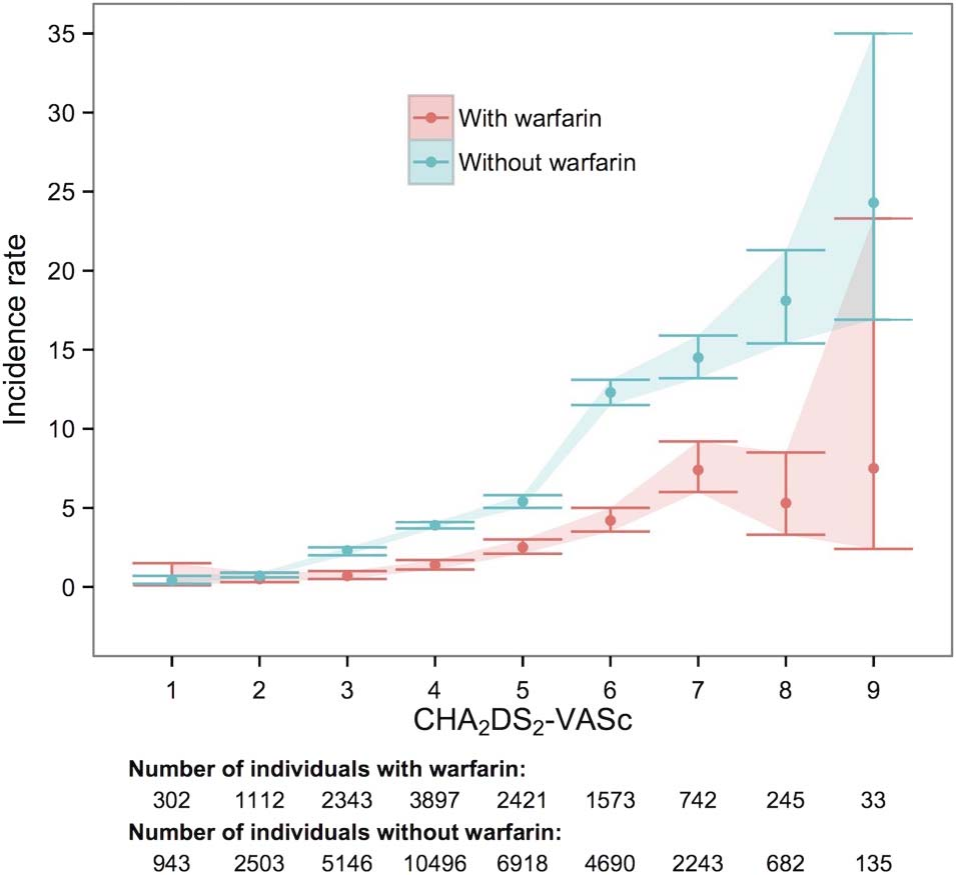
Incidence rates (95% CIs) per 100 person-years of ischaemic stroke in women by CHA_2_DS_2_-VASC scores, and use of warfarin. Individuals could contribute follow-up time to periods with and without warfarin.

One thousand and fifteen (14.5%) ISs occurred over 59 006 PYs of warfarin use and 5990 (85.5%) over 157 439 PYs of no warfarin use. IRs were lower with warfarin use (IR (95% CI)/100 PY: 1.7 (1.6 to 1.8) vs 3.8 (3.7 to 3.9), p value=0.00), with an IR ratio (95% CI) of 0.5 (0.4 to 0.5). For CHA_2_DS_2_-VASc=0, CHA_2_DS_2_-VASc=1 and CHA_2_DS_2_-VASc=2, IRs with versus without use of warfarin were (IR (95% CI)/100 PY: 0.4 (0.2 to 0.8) vs 0.2 (0.1 to 0.3), p value=0.16), (IR (95% CI)/100 PY: 0.4 (0.3 to 0.7) vs 0.7 (0.6 to 0.8), p value=0.03) and (IR (95% CI)/100 PY: 0.8 (0.7 to 1.0) vs 1.4 (1.3 to 1.6), p value=0.00). As figures 3 and 4 show IRs were lower with use of warfarin from CHA_2_DS_2_-VASc≥2 in men (IR (95% CI)/100 PY: 0.9 (0.7 to 1.1) vs 1.7 (1.5 to 1.9), p value=0.00), and from CHA_2_DS_2_-VASc≥3 in women (IR (95% CI)/100 PY: 0.7 (0.5 to 1.0) vs 2.3 (2.0 to 2.5), p value=0.00). IRs by sex and use of warfarin across all CHA_2_DS_2_-VASc scores are provided in table 3. IRs adjusted for propensity score quintiles were consistent with the unadjusted estimates (see online supplementary table S6).

**Table 3:** Incidence rates (95% CIs) per 100 person-years of ischaemic stroke by CHA_2_DS_2_-VASC scores, sex and use of warfarin

	With warfarin		Without warfarin			Population overall	
	Events	Rate	Events	Rate	p Value	Events	Rate
* CHA_2_DS_2_-VASc scores*							
Overall population							
0	7	0.4 (0.2 to 0.8)	21	0.2 (0.1 to 0.3)	0.23	28	0.2 (0.2 to 0.4)
1	27	0.4 (0.3 to 0.7)	126	0.7 (0.6 to 0.8)	0.03	153	0.6 (0.5 to 0.7)
2	87	0.8 (0.7 to 1.0)	366	1.4 (1.3 to 1.6)	0.00	453	1.2 (1.1 to 1.3)
3	144	1.0 (0.9 to 1.2)	869	2.6 (2.5 to 2.8)	0.00	1013	2.2 (2.0 to 2.3)
4	226	1.7 (1.5 to 2.0)	1447	4.0 (3.8 to 4.2)	0.00	1673	3.4 (3.2 to 3.6)
5	233	3.2 (2.8 to 3.6)	1183	6.2 (5.8 to 6.5)	0.00	1416	5.3 (5.1 to 5.6)
6	159	4.2 (3.6 to 4.8)	1201	12.1 (11.4 to 12.8)	0.00	1360	9.9 (9.4 to 10.4)
7	111	7.1 (5.9 to 8.6)	581	14.5 (13.4 to 15.7)	0.00	692	12.4 (11.5 to 13.4)
8	18	4.8 (3.0 to 7.6)	167	17.6 (15.1 to 20.5)	0.00	185	14.0 (12.1 to 16.2)
9	3	7.5 (2.4 to 23.3)	29	24.3 (16.9 to 35.0)	0.03	32	20.1 (14.2 to 28.4)
**0–9**	**1015**	**1.7 (1.6** to **1.8)**	**5990**	**3.8 (3.7** to **3.9)**	**0. 00**	**7005**	**3.2 (3.2** to **3.3)**
**Men**							
0	7	0.4 (0.2 to 0.8)	21	0.2 (0.1 to 0.3)	0.23	28	0.2 (0.2 to 0.4)
1	25	0.5 (0.3 to 0.7)	112	0.8 (0.6 to 0.9)	0.01	137	0.7 (0.6 to 0.8)
2	75	0.9 (0.7 to 1.1)	306	1.7 (1.5 to 1.9)	0.00	381	1.5 (1.3 to 1.6)
3	106	1.3 (1.1 to 1.5)	550	2.9 (2.7 to 3.2)	0.00	656	2.4 (2.2 to 2.6)
4	119	2.2 (1.8 to 2.6)	489	4.3 (3.9 to 4.7)	0.00	608	3.6 (3.3 to 3.9)
5	122	4.1 (3.4 to 4.9)	491	7.8 (7.1 to 8.5)	0.00	613	6.6 (6.1 to 7.1)
6	51	4.1 (3.1 to 5.4)	312	11.5 (10.3 to 12.9)	0.00	363	9.2 (8.3 to 10.1)
7	27	6.1 (4.3 to 9.2)	129	14.5 (12.2 to 17.3)	0.00	156	11.9 (10.1 to 13.9)
8	1	1.9 (0.3 to 13.3)	20	14.5 (9.4 to 22.5)	0.01	21	11.0 (7.2 to 16.8)
**0–8**	**533**	**1.6 (1.4** to **1.7)**	**2430**	**2.9 (2.8** to **3.1)**	**0.00**	**2963**	**2.5 (2.4** to **2.6)**
**Women**							
1	2	0.4 (0.1 to 1.5)	14	0.4 (0.2 to 0.7)	0.97	16	0.4 (0.3 to 0.7)
2	12	0.5 (0.3 to 0.9)	60	0.7 (0.6 to 0.9)	0.24	72	0.7 (0.5 to 0.8)
3	38	0.7 (0.5 to 1.0)	319	2.3 (2.0 to 2.5)	0.00	357	1.8 (1.6 to 2.0)
4	107	1.4 (1.1 to 1.7)	958	3.9 (3.7 to 4.1)	0.00	1065	3.3 (3.1 to 3.5)
5	111	2.5 (2.1 to 3.0)	692	5.4 (5.0 to 5.8)	0.00	803	4.7 (4.4 to 5.0)
6	108	4.2 (3.5 to 5.0)	889	12.3 (11.5 to 13.1)	0.00	997	10.2 (9.5 to 10.8)
7	84	7.4 (6.0 to 9.2)	452	14.5 (13.2 to 15.9)	0.00	536	12.6 (11.6 to 13.7)
8	17	5.3 (3.3 to 8.5)	147	18.1 (15.4 to 21.3)	0.00	164	14.5 (12.4 to 16.9)
9	3	7.5 (2.4 to 23.3)	29	24.3 (16.9 to 35.0)	0.03	32	20.1 (14.2 to 28.4)
**1–9**	**482**	**2.0 (1.8** to **2.1)**	**3560**	**4.8 (4.6** to **4.9)**	**0.00**	**4042**	**4.1 (3.9** to **4.2)**

#### NCB of warfarin

Nine hundred and six HSs occurred over 224 777 PYs. The overall NCB of warfarin was 1.9 (1.8 to 2.1) ISs avoided per 100 PYs. For CHA_2_DS_2_-VASc=0, CHA_2_DS_2_-VASc=1 and CHA_2_DS_2_-VASc=2, NCB was (NCB (95% CI)/100 PY: −0.3 (−0.8 to 0.1)), (NCB (95% CI)/100 PY: 0.1 (−0.2 to 0.4)) and (NCB (95% CI)/100 PY: 0.2 (−0.1 to 0.6)), respectively. A significant positive NCB was observed from CHA_2_DS_2_-VASc≥2 in men (NCB (95% CI)/100 PY: 0.5 (0.1 to 0.9)) and from CHA_2_DS_2_-VASc≥3 in women (NCB (95% CI)/100 PY: 1.5 (1.1 to 1.9)). NCB estimates across all CHA_2_DS_2_-VASc scores are provided in table 4.

**Table 4:** Net clinical benefit (95% CIs) per 100 person-years with warfarin, by CHA_2_DS_2_-VASC scores and sex

	Total stroke events		Net clinical benefit
	Ischaemic stroke	Haemorrhagic stroke	
*CHA_2_DS_2_-VASc scores*			
Overall population			
0	28	8	−0.3 (−0.8 to 0.1)
1	153	54	0.1 (−0.2 to 0.4)
2	453	95	0.2 (−0.1 to 0.6)
3	1013	165	1.5 (1.2 to 1.8)
4	1673	237	2.2 (1.8 to 2.6)
5	1416	180	3.2 (2.6 to 3.8)
6	1360	104	7.7 (6.7 to 8.8)
7	692	45	7.2 (5.2 to 9.1)
8	185	18	12.8 (8.9 to 16.9)
9	32	0	16.8 (1.8 to 31.5)
**0–9**	7005	906	1.9 (1.8 to 2.1)
Men			
0	28	8	−0.3 (−0.8 to 0.1)
1	137	48	0.1 (−0.2 to 0.4)
2	381	79	0.5 (0.1 to 0.9)
3	656	103	1.5 (1.1 to 1.9)
4	608	93	2.0 (1.3 to 2.7)
5	613	88	3.9 (2.6 to 4.9)
6	363	26	7.1 (5.2 to 9.1)
7	156	15	8.6 (4.7 to 13.0)
8	21	3	15.9 (7.0 to 25.7)
**0–8**	2963	463	1.2 (1.0 to 1.4)
Women			
1	16	6	0.3 (−0.4 to 0.8)
2	72	16	−0.1 (−0.6 to 0.3)
3	357	62	1.5 (1.1 to 1.9)
4	1065	144	2.4 (2.0 to 2.8)
5	803	92	3.1 (2.3 to 3.8)
6	997	78	8.0 (6.6 to 9.3)
7	536	30	6.8 (4.4 to 9.1)
8	164	15	12.4 (7.9 to 17.3)
9	32	0	16.8 (3.1 to 30.5)
**1–9**	4042	443	2.7 (2.4 to 3.0)

## Discussion

We conducted the first large-scale nationally representative study of the potential benefits and harms of warfarin in individuals with AF across stroke risk and across primary and secondary care, and had two major findings. First, we confirmed that CHA_2_DS_2_-VASc accurately stratifies stroke risk in individuals with initial record of diagnosis in primary and secondary care, however clinical information recorded in both primary and secondary care must be considered in order to correctly assign CHA_2_DS_2_-VASc scores. Second, in individuals who were truly CHA_2_DS_2_-VASc=1, the absolute risk of IS (0.4 (0.3 to 0.7) with warfarin, and 0.7 (0.6 to 0.8) without warfarin) was relatively low and similar to the original derivation cohort of the CHA_2_DS_2_-VASc score,^1^ and the NCB of warfarin was positive but non-significant (0.1 (−0.2 to 0.4)). We therefore found insufficient evidence to support anticoagulation with warfarin in individuals with CHA_2_DS_2_-VASc=1.

### Findings in context

Our IS IR of 0.7 (0.6 to 0.8) for CHA_2_DS_2_-VASc=1 without warfarin is low compared with previous reports (see online supplementary table S7) although consistent with the levels of uncertainty in a recent systematic review which reported an annual rate of 1.6% but with wide CIs (0% to 3.23%). Three lines of evidence from sensitivity analyses in men with CHA_2_DS_2_-VASc=1 (see online supplementary table S8) suggest that our estimates of stroke incidence are likely to be robust and offer reasons why they may differ from previous reports. First, as found in our main analysis, secondary care records underestimate stroke risk in half of individuals, and therefore previous studies which have predominantly focused on secondary care populations are likely to be biased by misclassification of CHA_2_DS_2_-VASc scores. In a sensitivity analysis of men with CHA_2_DS_2_-VASc=1 according to secondary care records (which included 53% who were truly CHA_2_DS_2_-VASc≥2), we found an IR of 1.4 (1.2 to 1.5), which is twice as high as the rate found for men who were truly CHA_2_DS_2_-VASc=1 (0.7 (0.6 to 0.8)) and similar to the meta-analysed rate found in the recent systematic review (1.6 (0 to 3.23)). Importantly, we confirm that if CHA_2_DS_2_-VASc risk is underestimated then stroke rates at the lower end of the CHA_2_DS_2_-VASc scale are overestimated, which has implications on treatment decisions. Second, Friberg, *et al*^25^ reported that variation in the literature also exists because of differences in the way stroke is defined, and that a 44% higher IR is observed when including wider thromboembolic end points. We also confirmed this in a sensitivity analysis, and found that for men with CHA_2_DS_2_-VASc=1, the IR doubled from 0.7 (0.6 to 0.8) to 1.4 (1.2 to 1.5) when systemic embolism, pulmonary embolism and transient ischaemic attacks were included as a composite end point. Third, differences in previously reported stroke rates at CHA_2_DS_2_-VASc=1 may exist because not all 1-point scoring risk factors (heart failure, hypertension, diabetes mellitus, vascular disease, age 65–74 years, female sex) confer the same stroke risk.^28^ In sensitivity analyses, we found that age 65–74 years conferred the highest stroke risk with IR of 1.2 (0.9 to 1.5). Thus, population-based IRs of CHA_2_DS_2_-VASc=1 will depend upon the distribution of 1-point scoring risk factors within the population.

Unlike the recent systematic review, our findings with regard to CHA_2_DS_2_-VASc=1 are supported by NCB analyses. To our knowledge, this is the first NCB analysis of warfarin to date that includes both individuals with initial record of diagnosis in primary and secondary care. We found a positive but non-significant treatment benefit of warfarin in individuals with CHA_2_DS_2_-VASc=1 (0.1 (−0.2 to 0.4)), and therefore insufficient evidence to support anticoagulation with warfarin in these individuals. Existing NCB analyses of warfarin have predominantly focused on individuals with initial record of diagnosis in secondary care, but have also shown an unclear benefit of treatment at CHA_2_DS_2_-VASc=1, including in both the nationwide Danish (−0.02 (−0.15 to 0.11)),^29^ and Swedish (0.00 (not reported))^30^ cohorts.

### Direct oral anticoagulants

A question that remains is whether the newer DOACs (dabigatran, rivaroxaban, apixaban and edoxaban) have a role in the treatment of lower-risk individuals. In DOAC trials (eg, randomized evaluation of long-term anticoagulant therapy (RE-LY),^31^ rivaroxaban once daily oral direct factor xa inhibition compared with vitamin k antagonism for prevention of stroke and embolism trial in atrial fibrillation (ROCKET-AF),^32^ apixaban for reduction in stroke and other thromboembolic events in atrial fibrillation (ARISTOTLE)^33^ and effective anticoagulation with factor xa next generation in atrial fibrillation-thrombolysis in myocardial Infarction 48 (ENGAGE AF-TIMI 48)^34^) all four agents were shown to be as effective in preventing ISs as warfarin, and associated with fewer HSs (see online supplementary table S9). We applied the trial reported relative risks of IS and HS to our data to consider the NCB of DOACs compared with no treatment. We found a significant positive NCB at CHA_2_DS_2_-VASc=1 across all agents (see online supplementary table S10), and therefore some, although extrapolated evidence that DOACs may be a more suitable treatment option for those at lower stroke risk. We caution against interpreting this finding too strongly, however, as the extrapolated model takes on multiple assumptions. First, it assumes that the relative risk reduction is constant over the entire period of follow-up, and across all levels of the CHA_2_DS_2_-VASc score. Second, it assumes that the trial population is representative of the general AF population. And third, it assumes that the benefit and harm end point definitions are equivalent. All four DOAC trials were approximately 2 years in duration, which is comparable to the 2.2 median years of follow-up in this analysis. However based on trial eligibility criteria, patients at CHA_2_DS_2_-VASc=1 were largely excluded from DOAC trials, and as previously demonstrated these trials represented only a half to two-thirds of the AF population in the UK.^35^ Lastly, benefit and harm end point definitions did vary across trials (see online supplementary table S9).

### Clinical implications

We confirmed that CHA_2_DS_2_-VASc is valid for estimating stroke risk in individuals with initial record of diagnosis in primary and secondary care, and therefore advocate use of the score across the full patient pathway. While we showed that lack of integration of primary and secondary care information may lead to inaccurate CHA_2_DS_2_-VASc scores, we do not regard this as an issue at point of care as clinicians are able to verify stroke risk factors with patients directly. However with the advent of clinical decision support systems,^36^ greater integration of primary and secondary care, and better recording of risk factors is urgently required in order to avoid undue patient harm through underestimation of stroke risk. Finally, we found insufficient evidence to support stroke prevention with warfarin at CHA_2_DS_2_-VASc=1, and this may have relevance for future treatment guidelines.^37^

### Research implications

Our findings highlight the value of linked EHRs in investigating individuals across the full pathway of primary and secondary care, and crucially in identifying individuals with CHA_2_DS_2_-VASc=1 in whom treatment guidelines have so far been unclear. Primary care records were instrumental in identifying individuals with CHA_2_DS_2_-VASc=1 and we therefore propose wider utilisation of existing and discovery of new primary care data sources for studying these individuals in future research, and in particular in ‘real-world’ comparative effectiveness studies of DOACs. By comparison, several large-scale secondary care data sources have already been used in AF research,^25^ ^28^ ^38^ however these are currently limited in terms of depth of clinical information, such as lack of prescriptions or biomarker data. We therefore propose wider linkage of routinely collected records to other clinical data sources such as disease-specific registries where deeper phenotype information is contained.

### Strengths and limitations

Our study's principal strength was the inclusion of individuals, risk factors and end points captured across the full pathway of primary and secondary care, which is unlike previous studies that have predominantly focused on secondary care populations. We were limited by the depth and completeness of clinical information currently recorded in primary and secondary care records, however, we minimised the impact of this by analysing multiple data sources, and adopting robust phenotypes for capturing individuals, risk factors and end points. An example of the value of CALIBER phenotypes comes from hypertension. As shown, the proportion of individuals with baseline hypertension rose significantly when information from diagnosis codes was supplemented with blood pressure measurements and prescriptions data. It is however still possible that some individuals, risk factors and end points may have been overlooked, but we consider the number affected to be minimal, and less than previous studies. While the lack of DOAC data may be considered a limitation of the present analysis, it should be noted that warfarin remains the most widely used oral anticoagulant. Therefore, studies involving pre-DOAC era cohorts such as this are still shown to be of contemporary relevance. Lastly, though we included a large sample size of 70 000 individuals, we found that only a quarter of the overall population had CHA_2_DS_2_-VASc scores 0, 1 and 2. We therefore cannot rule out the possibility that a positive NCB of warfarin may be observed at CHA_2_DS_2_-VASc=1 given a larger study population.

## Conclusion

CHA_2_DS_2_-VASc accurately stratifies IS risk in individuals with AF across both primary and secondary care. However IRs of IS at CHA_2_DS_2_-VASc=1 are lower than previously reported, which may change the decision to start anticoagulation with warfarin in these individuals.

**Key messages**

**What is already known on this subject?**

- It is unclear whether individuals with atrial fibrillation (AF) and CHA_2_DS_2_-VASc=1 benefit from stroke prevention with warfarin. Previous large-scale, population-based studies have reported incidence of ischaemic stroke (IS) in individuals with CHA_2_DS_2_-VASc=1; however have neglected the full patient pathway by focusing on those with initial record of diagnosis in secondary care.

**What might this study add?**

- We confirmed that CHA_2_DS_2_-VASc accurately stratifies IS risk in individuals with AF across both primary and secondary care, however incidence rates (IRs) at CHA_2_DS_2_-VASc=1 are lower than previously reported. We found a significant positive net clinical benefit of warfarin from CHA_2_DS_2_-VASc≥2 in men and from CHA_2_DS_2_-VASc≥3 in women, with 0.5 (0.1 to 0.9) and 1.5 (1.1 to 1.9) ISs avoided per 100 person-years of warfarin treatment, respectively.

**How might this impact on clinical practice?**

- IRs at CHA_2_DS_2_-VASc=1 are lower than previously reported, which may change the decision to start anticoagulation with warfarin in these individuals.
