# Supplementary material for: Net clinical benefit of warfarin in individuals with atrial fibrillation across stroke risk and across primary and secondary care
Source: Heart. 2016 Aug 31;103(3):210–8. doi: 10.1136/heartjnl-2016-309910 (PMC5284481; doi:10.1136/heartjnl-2016-309910)
Supplement: supplementary data [file heartjnl-2016-309910supp.pdf]

SUPPLEMENTARY MATERIAL

Net clinical benefit of warfarin in individuals with atrial fibrillation across stroke risk and across primary and secondary care.

Authors

Victoria Allan[1] Amitava Banerjee[1] Anoop Dinesh Shah[1] Riyaz Patel[1] Spiros Denaxas[1] Juan-Pablo Casas[1] Harry Hemingway

Affiliations

[1] Farr Institute of Health Informatics Research, Institute of Health Informatics, University College London, London, United Kingdom.

Contents

Table S1. Phenotype definition to exclude valvular atrial fibrillation 3
Table S2. CHA_2DS_2-VASc risk factors in individuals with and without use of warfarin 5
Table S3. CHA_2DS_2-VASc risk factors in men and women 6
Table S4. CHA_2DS_2-VASc risk factor completeness in primary and secondary care records 7
Figure S1. CHA_2DS_2-VASc risk factor completeness visualised in Venn diagrams 8
Table S5. CHA_2DS_2-VASc scores calculated using primary and secondary care records 9
Table S6. Propensity score adjusted incidence rates 10
Table S7. Supplementary evidence table comparing incidence rates in previous studies 11
Table S8. Sensitivity analyses in men with CHA_2DS_2-VASc=1 12
Table S9. Relative risks in direct oral anticoagulant trials 13
Table S10. Net clinical benefit of direct oral anticoagulants 14
Supplemental references 16

Table S1. Phenotype definition for valvular disease, which includes 45 Read codes, 9 International Classification of Diseases (ICD) codes, and 11 operation and procedure (OPCS) codes for mitral valve disease, rheumatic mitral regurgitation and prosthetic mitral, aortic or unspecified valve replacements.

Coding System	Code	Description
Read (medcode)	1267	Mitral valve diseases
Read (medcode)	1756	Replacement of aortic valve
Read (medcode)	1885	Mitral stenosis
Read (medcode)	3731	Replacement of mitral valve NEC
Read (medcode)	3911	Replacement of mitral valve
Read (medcode)	5643	Replacement of valve of heart NEC
Read (medcode)	6631	H/O: heart valve recipient
Read (medcode)	7276	Prosthetic replacement of valve of heart NEC
Read (medcode)	7894	Plastic repair of mitral valve
Read (medcode)	8274	Mitral and aortic stenosis
Read (medcode)	10078	Diseases of mitral and aortic valves
Read (medcode)	15133	Replacement of aortic valve NEC
Read (medcode)	16545	Rheumatic mitral valve disease
Read (medcode)	17257	Revision of plastic repair of mitral valve
Read (medcode)	17334	H/O: artificial heart valve
Read (medcode)	17596	Mitral stenosis and aortic regurgitation
Read (medcode)	17812	Open mitral valvotomy
Read (medcode)	18475	Combined disorders of mitral, aortic and tricuspid valves
Read (medcode)	19246	Closed mitral valvotomy
Read (medcode)	19390	Prosthetic replacement of mitral valve
Read (medcode)	19699	Disorders of both mitral and tricuspid valves
Read (medcode)	21807	Mitral incompetence - rheumatic
Read (medcode)	22582	Mitral valvuloplasty NEC
Read (medcode)	22837	Mitral regurgitation - rheumatic
Read (medcode)	24557	Mitral valve disorders NOS
Read (medcode)	28662	Nonrheumatic mitral valve stenosis
Read (medcode)	28871	[V]Has artificial heart valve
Read (medcode)	29158	Mitral and aortic valve disease NOS
Read (medcode)	29887	Replacement of unspecified valve of heart
Read (medcode)	30443	Mitral valve disease NOS
Read (medcode)	30567	Bjork-Shiley prosthetic replacement of mitral valve
Read (medcode)	32435	Rheumatic mitral stenosis
Read (medcode)	35812	Prosthetic replacement of aortic valve

Read (medcode)	36638	Mitral valvuloplasty
Read (medcode)	40264	[V]Presence of prosthetic heart valve
Read (medcode)	41168	Plastic repair of mitral valve NOS
Read (medcode)	44328	Mitral stenosis with regurgitation
Read (medcode)	44488	Mitral stenosis with insufficiency
Read (medcode)	49355	Mitral stenosis and aortic insufficiency
Read (medcode)	49592	Carpentier prosthetic replacement of mitral valve
Read (medcode)	51879	Rheumatic mitral insufficiency
Read (medcode)	53696	Percutaneous transluminal mitral valvotomy
Read (medcode)	57091	Congenital mitral stenosis
Read (medcode)	60957	Other specified plastic repair of mitral valve
Read (medcode)	61250	Mitral stenosis and aortic incompetence
ICD	I050	Mitral stenosis, rheumatic
ICD	I051	Rheumatic mitral insufficiency
ICD	I052	Mitral stenosis with insufficiency, rheumatic
ICD	I058	Other mitral valve diseases, rheumatic
ICD	I059	Mitral valve disease unspecified, rheumatic
ICD	I080	Disorders of both mitral and aortic valves, rheumatic
ICD	I081	Disorders of both mitral and tricuspid valves, rheumatic
ICD	I083	Combined disorders of mitral, aortic and tricuspid valves, rheumatic
ICD	I342	Nonrheumatic mitral (valve) stenosis
OPCS	K023	Implantation of prosthetic heart
OPCS	K253	Prosthetic replacement of mitral valve
OPCS	K254	Replacement of mitral valve NEC
OPCS	K258	Other specified plastic repair of mitral valve
OPCS	K259	Unspecified plastic repair of mitral valve
OPCS	K263	Prosthetic replacement of aortic valve
OPCS	K264	Replacement of aortic valve NEC
OPCS	K293	Prosthetic replacement of valve of heart NEC
OPCS	K294	Replacement of valve of heart NEC
OPCS	K301	Revision of plastic repair of mitral valve
OPCS	K311	Open mitral valvotomy

Table S2. Comparison of baseline CHA_2DS_2-VASc risk factors in individuals with and without use of warfarin.

	With warfarin		Without warfarin		Overall	
**Number of individuals**	**30 067**		**40 139**		**70 206**	
	**N**	**%**	**N**	**%**	**N**	**%**
**C**ongestive heart failure	10400	25.9	7032	23.4	17432	24.8
**H**ypertension	32641	81.3	25122	83.6	57763	82.3
Diagnosis	23973	59.7	17916	59.6	41889	59.7
Blood pressure medication	28657	71.4	22486	74.8	51143	72.9
Blood pressure measures	22060	55.0	17550	58.4	39610	56.4
**A**ge ≥ 75 [**2**]	27235	67.9	14955	49.7	42190	60.1
**D**iabetes	5746	14.3	4243	14.1	9989	14.2
**S**troke/TIA/systemic embolism [**2**]	7506	18.7	5319	17.7	12825	18.3
**V**ascular disease	7935	19.8	5897	19.6	13832	19.7
Myocardial infarction	5439	13.6	4146	13.8	9585	13.7
Peripheral vascular disease	3341	8.3	2362	7.9	5703	8.1
**A**ge 65 to 74	6975	17.4	9321	31.0	16296	23.2
**S**ex **C**ategory [female]	21618	63.0	12668	37.0	34286	48.8
**CHA_2DS_2-VASc scores**						
0	1414	3.5	1072	3.6	2486	3.5
1	2786	6.9	2851	9.5	5637	8.0
2	4485	11.2	4854	16.1	9339	13.3
3	7309	18.2	6461	21.5	13770	19.6
4	10102	25.2	6808	22.6	16910	24.1
5	6994	17.4	4232	14.1	11226	16.0
6	4383	10.9	2389	8.0	6772	9.7
7	1995	5.0	1070	3.6	3065	4.4
8	566	1.4	297	1.0	863	1.2
9	105	0.3	33	0.1	138	0.2

Table S3. Comparison of baseline CHA_2DS_2-VASc risk factors in men and women.

	**Men**		**Women**		**Overall**	
**Number of individuals**	**35 920**		**34 286**		**70 206**	
	**N**	**%**	**N**	**%**	**N**	**%**
**C**ongestive heart failure	8458	23.6	8974	26.2	17432	24.8
**H**ypertension	28350	78.9	29413	85.8	57763	82.3
Diagnosis	19702	54.9	22187	64.7	41889	59.7
Blood pressure medication	24784	69	26359	76.9	51143	72.9
Blood pressure measures	18794	52.3	20816	60.7	39610	56.4
**A**ge ≥ 75 [**2**]	17828	49.6	24362	71.1	42190	60.1
**D**iabetes	5545	15.4	4444	13.0	9989	14.2
**S**troke/TIA/systemic embolism [**2**]	6136	17.1	6689	19.5	12825	18.3
**V**ascular disease	8435	23.5	5397	15.7	13832	19.7
Myocardial infarction	6120	17.0	3465	10.1	9585	13.7
Peripheral vascular disease	3292	9.2	2411	7.0	5703	8.1
**A**ge 65–74	9861	27.5	6435	18.8	16296	23.2
**S**ex **C**ategory [female]	0	0.0	34286	100	34286	48.8
**CHA_2DS_2-VASc scores**						
0	2486	6.9	0	0.0	2486	3.5
1	4690	13.1	947	2.8	5637	8.0
2	6813	19.0	2526	7.4	9339	13.3
3	8562	23.8	5208	15.2	13770	19.6
4	6279	17.5	10631	31.0	16910	24.1
5	4168	11.6	7058	20.6	11226	16.0
6	1991	5.5	4781	13.9	6772	9.7
7	775	2.2	2290	6.7	3065	4.4
8	156	0.4	707	2.1	863	1.2
9	0	0.0	138	0.4	138	0.2

Table S4. Completeness of recording CHA_2DS_2-VASc risk factors in primary care records, and secondary care records.

	Total individuals with risk factor	Individuals with risk factor recorded in primary care		Individuals with risk factor recorded in secondary care	
	**N**	**N**	**%**	**N**	**%**
Congestive heart failure	17432	12043	69.1	11145	63.9
Hypertension	57763	57197	99.0	23310	40.4
Diabetes mellitus	9989	9339	93.5	7366	73.7
Stroke/ TIA / systemic embolism	12825	10671	83.2	6171	48.1
Vascular disease	13832	11956	86.4	6419	46.4

Legend

Total individuals with risk factor refers to total number of individuals with recorded diagnosis of the risk factor, as recorded in either primary or secondary care records. Individuals with risk factor recorded in primary care refers to the 'completeness' of recording the risk factor in primary records, where 100% indicates absolute completeness. For example 99% of total individuals with hypertension, had a record of hypertension in primary care records. Individuals with risk factor recorded in secondary care refers to the 'completeness' of recording the risk factor in secondary records.

Figure S1. Venn diagrams comparing numbers of CHA_2DS_2-VASc risk factors captured in primary care, secondary care and in both sources linked. Venn circles are scaled according to the proportion of individuals that they represent.

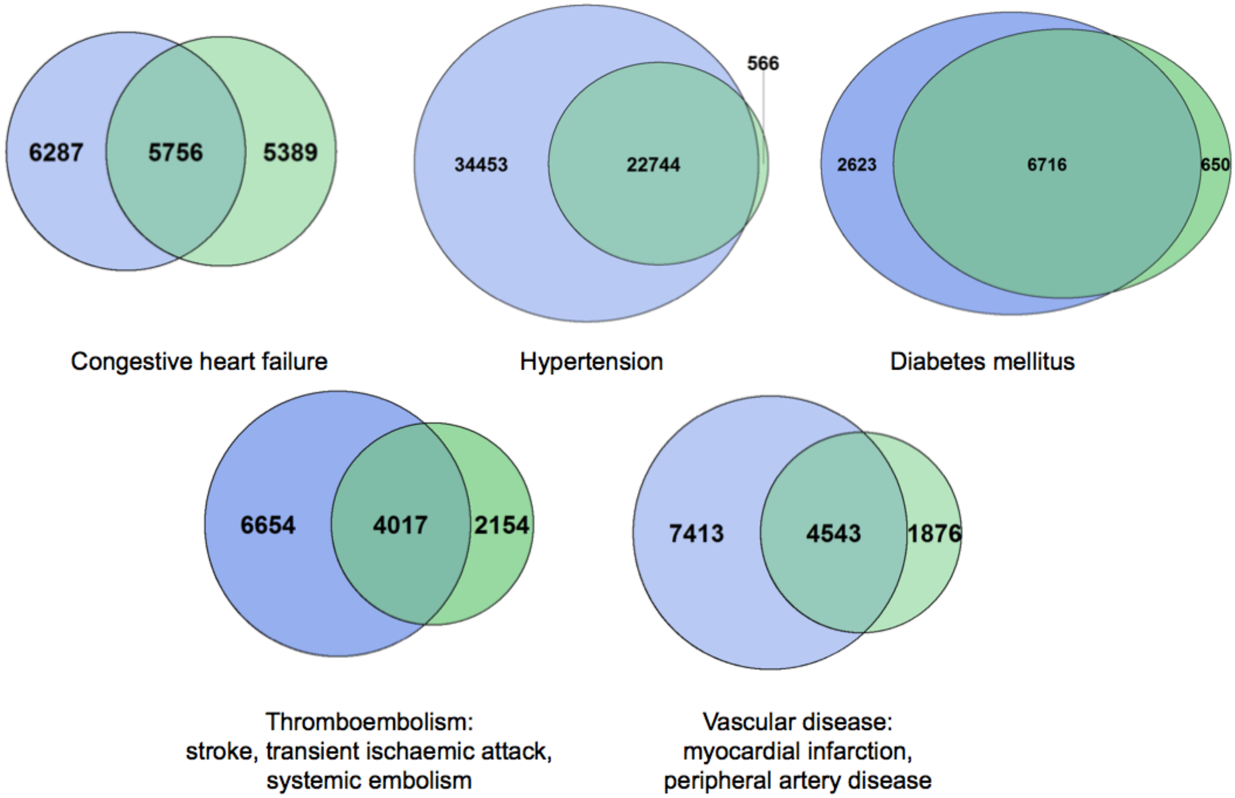

Table S5. CHA_2DS_2-VASc scores based on primary and secondary care records, compared to both data sources linked.

	Secondary care records only		Primary–secondary care records	
**Individuals with initial record of diagnosis in secondary care**	**40 638**		**40 638**	
**CHA_2DS_2-VASc scores**	**N**	**%**	**N**	**%**
0	2156	5.3	1181	2.9
1	4042	10.0	2665	6.6
2	7196	17.7	4519	11.1
3	10309	25.4	7107	17.5
4	8,768	21.6	9578	23.6
5	4,794	11.8	7514	18.5
6	2,361	5.8	4906	12.1
7	776	1.9	2341	5.8
8	201	0.5	707	1.7
9	35	0.1	120	0.3

	Primary care records only		Primary–secondary care records	
**Individuals with initial record of diagnosis in primary care**	**29 568**		**29 568**	
**CHA_2DS_2-VASc scores**	**N**	**%**	**N**	**%**
0	1320	4.5	1305	4.4
1	3041	10.3	2972	10.1
2	4889	16.5	4820	16.3
3	6838	23.1	6663	22.5
4	7450	25.2	7332	24.8
5	3537	12.0	3712	12.6
6	1750	5.9	1866	6.3
7	611	2.1	724	2.5
8	121	0.4	156	0.5
9	11	0.0	18	0.1

Table S6. Propensity score adjusted incidence rates [95% confidence intervals] per 100 person-years of ischaemic stroke by CHA_2DS_2-VASC scores, sex, and use of warfarin

	With warfarin		Without warfarin		
	**Predicted events**	**Adjusted rate**	**Predicted events**	**Adjusted rate**	**P-value**
**CHA_2DS_2-VASc scores**					
**Overall population**					
0	7	0.4 [0.1,0.7]	23	0.2 [0.1,0.3]	0.31
1	27	0.4 [0.3,0.6]	127	0.7 [0.6,0.8]	0.03
2	89	0.8 [0.6,1.0]	355	1.4 [1.2,1.5]	0.00
3	152	1.1 [0.9,1.3]	816	2.5 [2.3,2.6]	0.00
4	236	1.8 [1.6,2.0]	1366	3.8 [3.6,4.0]	0.00
5	236	3.2 [2.8,3.6]	1129	5.9 [5.6,6.2]	0.00
6	170	4.4 [3.7,5.1]	1154	11.6 [11.0,12.2]	0.00
7	116	7.4 [6.1,8.8]	553	13.8 [12.7,14.8]	0.00
8	19	5.0 [2.7,7.3]	167	17.6 [15.2,20.0]	0.00
9	3	8.7 [0.4,16.9]	30	24.9 [17.0,32.7]	0.03
**Men**					
0	7	0.4 [0.1,0.7]	23	0.2 [0.1,0.3]	0.31
1	25	0.4 [0.3,0.6]	110	0.7 [0.6,0.9]	0.02
2	78	0.9 [0.7,1.1]	306	1.7 [1.5,1.9]	0.00
3	114	1.4 [1.1,1.6]	542	2.9 [2.6,3.1]	0.00
4	171	3.2 [2.3,4.1]	552	4.8 [4.3,5.3]	0.00
5	153	5.1 [3.8,6.5]	580	9.2 [8.2,10.1]	0.00
6	74	5.9 [1.8,10.0]	425	15.7 [13.5,17.9]	0.00
7	39	9.1 [1.4,16.7]	177	19.9 [15.5,24.3]	0.00
8	1	1.1 [0.0,3.0]	28	20.6 [9.7,31.4]	0.00
**Women**					
1	1	0.1 [0.0,0.4]	13	0.4 [0.1,0.7]	0.54
2	7	0.3 [0.1,0.4]	42	0.5 [0.3,0.6]	0.17
3	38	0.7 [0.5,0.9]	271	1.9 [1.7,2.2]	0.00
4	102	1.3 [1.1,1.6]	815	3.3 [3.1,3.5]	0.00
5	109	2.5 [2.0,2.9]	610	4.8 [4.4,5.2]	0.00
6	107	4.1 [3.3,4.9]	791	10.9 [10.2,11.7]	0.00
7	82	7.3 [5.7,8.8]	397	12.7 [11.5,13.9]	0.00
8	17	5.3 [2.8,7.7]	140	17.3 [14.8,19.9]	0.00
9	3	8.7 [0.4,16.9]	30	24.9 [17.0,32.7]	0.03

Legend: Incidence rates were adjusted for propensity score quintiles. Propensity score was generated using a logistic regression model, which predicted probability of warfarin use (yes, no), based on CHA_2DS_2-VASC risk factors, age at initial record of diagnosis of atrial fibrillation, and source of initial record of atrial fibrillation (primary, or secondary care).

Table S7. Supplementary evidence table comparing studies that reported incidence rates for ischaemic stroke in atrial fibrillation patients with CHA_2DS_2-VASc=1 [including 1 in men | 2 in women], sorted by estimate size.

		Data source				Endpoints						Risk group		Incidence Rate [95% CI] per 100 PY		
Author, year	Country	PC	SC	Total patients	Maximum follow-up	IS	US	SE	PE	TIA	Total events	CHA_2DS_2-VASc	Sex	Anticoagulants	No anticoagulants	Ref.
Huang, 2014	China	○	●	358	4.5	●	○	○	○	○	70	1	men	NR [NR]	6.60 [NR]	[1]
Chao, 2015	Taiwan	○	●	12935	6.0	●	○	○	○	○	1858	1	men	NR [NR]	2.75 [NR]	[2]
Chao, 2015	Taiwan	○	●	7900	6.0	●	○	○	○	○	1174	2	women	NR [NR]	2.55 [NR]	[2]
Olesen, 2011	Denmark	○	●	8203	1.0	●	○	●	●	○	NR	1	both	NR [NR]	2.01 [1.70,2.36]	[3]
Olesen, 2012	Denmark	○	●	10062	12.0	●	○	●	○	●	159	1	both	NR [NR]	1.79 [1.53,2.09]	[4]
Olesen, 2011	Denmark	○	●	14515	1.0	●	○	●	○	●	256	1	both	1.28 [1.02,1.61]	1.62 [1.37,1.92]	[5]
Lip, 2015	Denmark	○	●	15860	1.0	●	○	●	○	○	188	1<br>2	men<br>women	1.06 [NR]	1.55 [NR]	[6]
Olesen, 2011	Denmark	○	●	8203	5.0	●	○	●	●	○	NR	1	both	NR [NR]	1.51 [1.37,1.67]	[3]
Lip, 2015	Denmark	○	●	15860	1.0	●	○	○	○	○	182	1<br>2	men<br>women	1.02 [NR]	1.50 [NR]	[6]
Olesen, 2011	Denmark	○	●	8203	10.0	●	○	●	●	○	NR	1	both	NR [NR]	1.45 [1.32,1.58]	[3]
Olesen, 2012	Denmark	○	●	10062	12.0	●	○	●	○	●	662	1	both	NR [NR]	1.44 [1.34,1.56]	[4]
Lip, 2015	Denmark	○	●	15860	4.5	●	○	●	○	○	987	1<br>2	men<br>women	1.08 [NR]	1.24 [NR]	[6]
Lip, 2015	Denmark	○	●	15860	4.5	●	○	○	○	○	936	1<br>2	men<br>women	1.02 [NR]	1.18 [NR]	[6]
Friberg, 2012	Sweden	○	●	6770	3.5	●	●	●	○	●	NR	1	both	NR [NR]	0.90 [NR]	[7]
Guo, 2012	China	○	●	114	3.0	●	○	●	●	○	NR	1	both	NR [NR]	0.90 [NR]	[8]
**CALIBER, 2016**	**England**	**●**	**●**	**5637**	**12.0**	**●**	**●**	**●**	**●**	**●**	**153**	**1**	**both**	**0.4 [0.3,0.7]**	**0.7 [0.6,0.8]**	
Lip, 2010	Europe	○	●	162	1.0	●	○	●	●	○	1	1	both	NR [NR]	0.60 [0.00,3.40]	[9]
Friberg, 2012	Sweden	○	●	6770	3.5	●	○	○	○	○	NR	1	both	NR [NR]	0.60 [NR]	[7]
Friberg, 2015	Sweden	○	●	NR	5.0	●	○	○	○	○	NR	1	men	NR [NR]	0.50–0.70	[10]
Forslund, 2014	Sweden	●	●	6682	1.0	●	○	○	○	○	NR	≤1	both	0.00–0.30	0.30–0.50	[11]
Friberg, 2015	Sweden	○	●	NR	5.0	●	○	○	○	○	NR	1	women	NR [NR]	0.10–0.20	[10]

Legend
CI – confidence interval, PY – person-years, PC –primary care, SC – secondary care, IS - ischaemic stroke, US – unclassified stroke, SE – systemic embolism, PE – pulmonary embolism, TIA - transient ischaemic attack, NR – not reported, ○ – no, ● – yes. Example: Huang, 2014. utilised secondary care data for identifying patients, risk factors, and endpoints, and included ischaemic stroke in the endpoint definition.

Table S8. Sensitivity analyses in men with CHA_2DS_2-VASc=1.

	Total patients	Initial AF diagnosis		CHA_2DS_2-VASc		Endpoint			Endpoint Definition					Total events	Incidence Rate [95% CI] per 100 PY
		PC	SC	PC	SC	PC	SC	DR	IS	US	SE	PE	TIA		
**By data source used for CHA_2DS_2-VASc:**															
Secondary care	3015[a]	○	●	○	●	●	●	●	●	●	○	○	○	156	**1.4 [1.2,1.6]**
Primary care	2563[a]	●	○	●	○	●	●	●	●	●	○	○	○	72	0.6 [0.5,0.8]
Total	4690	●	●	●	●	●	●	●	●	●	○	○	○	137	0.7 [0.6,0.8]
**By data source used for initial AF diagnosis:**															
Secondary care	2185	○	●	●	●	●	●	●	●	●	○	○	○	68	0.8 [0.6,1.0]
Primary care	2505	●	○	●	●	●	●	●	●	●	○	○	○	69	0.6 [0.5,0.7]
Total	4690	●	●	●	●	●	●	●	●	●	○	○	○	137	0.7 [0.6,0.8]
**By data source for endpoint:**															
Death register	760[b]	●	●	●	●	○	○	●	●	●	○	○	○	14	0.6 [0.4,1.1]
Primary care	4690	●	●	●	●	○	●	○	●	●	○	○	○	106	0.5 [0.4,0.6]
Secondary care	4690	●	●	●	●	●	○	○	●	●	○	○	○	90	0.4 [0.4,0.5]
Total	4690	●	●	●	●	●	●	●	●	●	○	○	○	137	0.7 [0.6,0.8]
**By endpoint definition:**															
Composite endpoint	4690	●	●	●	●	●	●	●	●	●	●	●	●	274	**1.4 [1.2,1.5]**
Primary endpoint	4690	●	●	●	●	●	●	●	●	●	○	○	○	137	0.7 [0.6,0.8]
**By use of warfarin:**															
Without warfarin	4629[c]	●	●	●	●	●	●	●	●	●	○	○	○	112	0.8 [0.6,0.9]
With warfarin	2549[c]	●	●	●	●	●	●	●	●	●	○	○	○	25	0.4 [0.3,0.7]
Total	4690	●	●	●	●	●	●	●	●	●	○	○	○	137	0.7 [0.6,0.8]
**By risk score component:**															
Age 65 to 74	1336	●	●	●	●	●	●	●	●	●	○	○	○	68	**1.2 [0.9,1.5]**
Hypertension	3053	●	●	●	●	●	●	●	●	●	○	○	○	66	0.5 [0.4,0.6]
Heart failure	126	●	●	●	●	●	●	●	●	●	○	○	○	2	0.5 [0.1,1.8]
Vascular disease	110	●	●	●	●	●	●	●	●	●	○	○	○	1	0.2 [0.0,1.5]
Diabetes mellitus	65	●	●	●	●	●	●	●	●	●	○	○	○	0	No events
Total	4690	●	●	●	●	●	●	●	●	●	○	○	○	137	0.7 [0.6,0.8]

Legend

Abbreviations as per table S6, and DR – death records.

[a] Includes individuals reclassified CHA2DS2-VASc ≥2 when stroke risk calculated from linked records,

[b] restricted to 760 all-cause deaths during follow-up, [c] individuals could contribute follow-up time to periods with and without warfarin

Table S9. Relative risks of ischaemic and haemorrhagic stroke in direct oral anticoagulant trials

			Ischaemic stroke		Haemorrhagic stroke	
**Ref**	**Trial**	**Follow-up**	**Definition**	**Relative risk**	**Definition**	**Relative risk**
[12]	**RE-LY**					
	Dabigatran 110mg	2.0 years	Ischaemic or unspecified stroke	1.11 [0.89,1.40]	Haemorrhagic stroke	0.31 [0.17,0.56]
	Dabigatran 150mg	2.0 years	Ischaemic or unspecified stroke	0.76 [0.60,0.98]	Haemorrhagic stroke	0.26 [0.14,0.49]
[13]	**ROCKET AF**					
	Rivaroxaban as treated	1.9 years	Stroke or systemic	0.79 [0.66,0.96]	Intracranial haemorrhage	0.67 [0.47,0.93]
	Rivaroxaban intention to treat	1.9 years	Stroke or systemic	0.88 [0.75,1.03]	Intracranial haemorrhage	0.67 [0.47,0.93]
[14]	**ARISTOTLE**					
	Apixaban	1.8 years	Ischaemic or uncertain type of stroke	0.92 [0.74,1.13]	Haemorrhagic stroke	0.51 [0.35,0.75]
[15]	**ENGAGE AF-TIMI 48**					
	Edoxaban 30mg	2.8 years	Ischaemic stroke	1.41 [1.19,1.67]	Haemorrhagic stroke	0.33 [0.22,0.50]
	Edoxaban 60mg	2.8 years	Ischaemic stroke	1.00 [0.83,1.19]	Haemorrhagic stroke	0.54 [0.38,0.77]

Table S10. Net clinical benefit [95% confidence intervals] per 100 person-years of warfarin or direct oral anticoagulants compared to no treatment, by CHA_2DS_2-VASC scores, and sex.

	Total stroke events		Net clinical benefit of oral anticoagulants vs. no treatment							
	Ischaemic stroke	Haemorrhagic stroke	Warfarin	Dabigatran 110mg	Dabigatran 150mg	Rivaroxaban AT	Rivaroxaban ITT	Apixaban	Edoxaban 30mg	Edoxaban 60mg
**CHA_2DS_2-VASc:**										
**Overall population**										
0	28	8	-0.3 [-0.8,0.1]	-0.2 [-0.6,0.1]	0.0 [-0.3,0.2]	-0.2 [-0.5,0.1]	-0.2 [-0.6,0.1]	-0.2 [-0.5,0.1]	-0.3 [-0.8,0.1]	-0.2 [-0.6,0.1]
1	153	54	0.1 [-0.2,0.4]	0.4 [0.1,0.6]	0.5 [0.3,0.7]	0.3 [0.1,0.6]	0.3 [0.1,0.5]	0.4 [0.1,0.6]	0.2 [-0.1,0.5]	0.3 [0.1,0.5]
2	453	95	0.2 [-0.1,0.6]	0.6 [0.3,0.9]	0.9 [0.7,1.1]	0.6 [0.4,0.9]	0.5 [0.3,0.8]	0.6 [0.4,0.9]	0.3 [0.0,0.6]	0.5 [0.3,0.8]
3	1013	165	1.5 [1.2,1.8]	1.8 [1.5,2.0]	2.2 [1.9,2.4]	1.9 [1.6,2.1]	1.8 [1.5,2.1]	1.8 [1.6,2.1]	1.4 [1.1,1.7]	1.7 [1.5,2.0]
4	1673	237	2.2 [1.8,2.6]	2.5 [2.2,2.9]	3.2 [2.9,3.4]	2.8 [2.5,3.1]	2.7 [2.3,3.0]	2.7 [2.4,3.0]	2.0 [1.6,2.4]	2.6 [2.2,2.9]
5	1416	180	3.2 [2.6,3.8]	3.4 [2.8,4.0]	4.6 [4.1,5.1]	4.1 [3.6,4.7]	3.8 [3.3,4.4]	3.9 [3.3,4.4]	2.5 [1.8,3.1]	3.6 [3.0,4.2]
6	1360	104	7.7 [6.7,8.8]	8.1 [7.0,9.1]	9.6 [8.7,10.5]	9.0 [8.0,9.9]	8.6 [7.6,9.6]	8.6 [7.6,9.6]	6.8 [5.6,8.0]	8.3 [7.2,9.3]
7	692	45	7.2 [5.2,9.1]	7.3 [5.1,9.3]	9.8 [8.1,11.6]	9.1 [7.4,10.8]	8.5 [6.7,10.3]	8.4 [6.5,10.2]	5.1 [2.6,7.5]	7.8 [5.8,9.7]
8	185	18	12.8 [8.9,16.9]	13.6 [9.7,17.5]	15.4 [12.0,19.0]	14.5 [10.8,18.2]	14.0 [10.3,17.9]	14.1 [10.5,18.0]	12.1 [7.8,16.4]	13.7 [10.0,17.6]
9	32	0	16.8 [1.8,31.5]	16.0 [0.3,31.3]	18.6 [5.8,32.5]	18.4 [5.2,32.4]	17.7 [3.6,32.0]	17.4 [3.0,31.7]	13.7 [-4.3,30.0]	16.8 [1.8,31.5]
**0-9**	7005	906	1.9 [1.8,2.1]	2.2 [2.1,2.4]	2.9 [2.7,3.0]	2.5 [2.4,2.7]	2.4 [2.2,2.5]	2.4 [2.3,2.6]	1.7 [1.5,1.9]	2.3 [2.1,2.4]
**Men**										
0	28	8	-0.3 [-0.8,0.1]	-0.1 [-0.6,0.1]	0.0 [-0.3,0.2]	-0.2 [-0.5,0.1]	-0.2 [-0.6,0.1]	-0.2 [-0.5,0.1]	-0.3 [-0.8,0.1]	-0.2 [-0.6,0.1]
1	137	48	0.1 [-0.2,0.4]	0.5 [0.1,0.7]	0.6 [0.4,0.8]	0.4 [0.1,0.6]	0.3 [0.1,0.6]	0.4 [0.1,0.6]	0.3 [-0.1,0.6]	0.3 [0.1,0.6]
2	381	79	0.5 [0.1,0.9]	1.0 [0.6,1.2]	1.2 [0.9,1.5]	0.9 [0.6,1.2]	0.8 [0.5,1.1]	0.9 [0.6,1.2]	0.6 [0.2,1.0]	0.8 [0.5,1.1]
3	656	103	1.5 [1.1,1.9]	2.0 [1.4,2.2]	2.3 [1.9,2.6]	2.0 [1.6,2.3]	1.8 [1.5,2.2]	1.9 [1.5,2.3]	1.4 [0.9,1.8]	1.8 [1.4,2.2]
4	608	93	2.0 [1.3,2.7]	2.8 [1.7,3.0]	3.2 [2.6,3.7]	2.8 [2.2,3.3]	2.6 [1.9,3.2]	2.6 [2.0,3.2]	1.7 [0.9,2.4]	2.4 [1.8,3.0]
5	613	88	3.9 [2.6,4.9]	5.0 [3.1,5.3]	5.7 [4.7,6.7]	5.1 [4.1,6.1]	4.7 [3.7,5.7]	4.8 [3.7,5.8]	3.0 [1.6,4.2]	4.4 [3.3,5.5]
6	363	26	7.1 [5.2,9.1]	8.3 [5.6,9.4]	8.9 [7.3,10.7]	8.4 [6.6,10.1]	8.0 [6.2,9.8]	8.0 [6.2,9.8]	6.2 [4.0,8.4]	7.6 [5.8,9.5]
7	156	15	8.6 [4.7,13.0]	10.1 [4.8,13.2]	11.1 [7.7,15.0]	10.4 [6.9,14.3]	9.8 [6.2,13.9]	9.8 [6.1,13.9]	6.9 [2.3,11.6]	9.2 [5.4,13.5]
8	21	3	15.9 [7.0,25.7]	16.0 [6.4,25.7]	16.3 [8.0,25.8]	16.3 [7.8,25.8]	16.1 [7.4,25.8]	16.0 [7.3,25.7]	15.1 [5.0,25.7]	15.9 [7.0,25.7]
**0-8**	2963	463	1.2 [1.0,1.4]	1.8 [1.3,1.7]	2.1 [1.9,2.3]	1.8 [1.6,1.9]	1.6 [1.4,1.8]	1.7 [1.5,1.9]	1.0 [0.8,1.3]	1.5 [1.3,1.7]

Table S10 (continued). Net clinical benefit [95% confidence intervals] per 100 person-years of warfarin or direct oral anticoagulants compared to no treatment, by CHA_2DS_2-VASC scores, and sex.

	Total stroke events		Net clinical benefit of oral anticoagulants vs. no treatment							
	Ischaemic stroke	Haemorrhagic stroke	Warfarin	Dabigatran		Rivaroxaban		Apixaban	Edoxaban	
				110mg	150mg	AT	ITT		30mg	60mg
**CHA_2DS_2-VASc:**										
**Women**										
1	16	6	0.3 [-0.4,0.8]	0.3 [-0.5,0.8]	0.4 [-0.2,0.8]	0.4 [-0.2,0.8]	0.3 [-0.3,0.8]	0.3 [-0.3,0.8]	0.1 [-0.8,0.8]	0.3 [-0.4,0.8]
2	72	16	-0.1 [-0.6,0.3]	0.3 [-0.2,0.5]	0.4 [0.0,0.7]	0.1 [-0.2,0.5]	0.1 [-0.3,0.5]	0.2 [-0.2,0.5]	0.0 [-0.5,0.4]	0.1 [-0.3,0.5]
3	357	62	1.5 [1.1,1.9]	1.9 [1.4,2.2]	2.0 [1.7,2.4]	1.8 [1.4,2.2]	1.8 [1.4,2.1]	1.8 [1.5,2.2]	1.6 [1.1,2.0]	1.7 [1.4,2.1]
4	1065	144	2.4 [2.0,2.8]	3.0 [2.4,3.1]	3.3 [2.9,3.6]	2.9 [2.6,3.3]	2.8 [2.4,3.2]	2.9 [2.5,3.2]	2.3 [1.9,2.8]	2.7 [2.4,3.1]
5	803	92	3.1 [2.3,3.8]	3.7 [2.5,3.9]	4.1 [3.6,4.7]	3.8 [3.2,4.4]	3.6 [2.9,4.2]	3.6 [2.9,4.2]	2.5 [1.6,3.3]	3.4 [2.7,4.0]
6	997	78	8.0 [6.6,9.3]	9.2 [7.0,9.6]	9.8 [8.7,11.0]	9.2 [8.0,10.4]	8.9 [7.6,10.1]	8.9 [7.6,10.1]	7.0 [5.5,8.5]	8.5 [7.2,9.8]
7	536	30	6.8 [4.4,9.1]	8.2 [4.5,9.1]	9.4 [7.5,11.4]	8.7 [6.7,10.8]	8.0 [6.0,10.2]	7.9 [5.9,10.1]	4.5 [1.8,7.3]	7.3 [5.1,9.6]
8	164	15	12.4 [7.9,17.3]	14.3 [8.8,18.0]	15.2 [11.4,19.5]	14.2 [10.3,18.8]	13.7 [9.7,18.4]	13.9 [9.8,18.4]	11.7 [6.6,16.8]	13.4 [9.1,18.0]
9	32	0	16.8 [3.1,30.5]	17.5 [1.5,30.3]	18.6 [6.6,31.3]	18.4 [6.1,31.2]	17.7 [4.8,30.9]	17.4 [4.2,30.8]	13.7 [-3.9,29.3]	16.8 [3.1,30.5]
**1-9**	4042	443	2.7 [2.4,3.0]	3.4 [2.7,3.3]	3.7 [3.5,3.9]	3.4 [3.1,3.6]	3.2 [2.9,3.5]	3.2 [3.0,3.5]	2.4 [2.1,2.7]	3.0 [2.8,3.3]

Legend

AT – as treated, ITT – intention to treat.

Supplemental references

1 Huang D, Anguo L, Yue WS, et al. Refinement of ischemic stroke risk in patients with atrial fibrillation and CHA2 DS2 -VASc score of 1. *Pacing and clinical electrophysiology : PACE* 2014;**37**:1442-7.

2 Chao TF, Liu CJ, Wang KL, et al. Should atrial fibrillation patients with 1 additional risk factor of the CHA2DS2-VASc score (beyond sex) receive oral anticoagulation? *Journal of the American College of Cardiology* 2015;**65**:635-42.

3 Olesen JB, Lip GY, Hansen ML, et al. Validation of risk stratification schemes for predicting stroke and thromboembolism in patients with atrial fibrillation: nationwide cohort study. *BMJ (Clinical research ed)* 2011;**342**:d124.

4 Olesen JB, Torp-Pedersen C, Hansen ML, et al. The value of the CHA2DS2-VASc score for refining stroke risk stratification in patients with atrial fibrillation with a CHADS2 score 0-1: a nationwide cohort study. *Thrombosis and haemostasis* 2012;**107**:1172-9.

5 Olesen JB, Lip GY, Lindhardsen J, et al. Risks of thromboembolism and bleeding with thromboprophylaxis in patients with atrial fibrillation: A net clinical benefit analysis using a 'real world' nationwide cohort study. *Thrombosis and haemostasis* 2011;**106**:739-49.

6 Lip GY, Skjoth F, Rasmussen LH, et al. Oral anticoagulation, aspirin, or no therapy in patients with nonvalvular AF with 0 or 1 stroke risk factor based on the CHA2DS2-VASc score. *Journal of the American College of Cardiology* 2015;**65**:1385-94.

7 Friberg L, Rosenqvist M, Lip GY. Evaluation of risk stratification schemes for ischaemic stroke and bleeding in 182 678 patients with atrial fibrillation: the Swedish Atrial Fibrillation cohort study. *European heart journal* 2012;**33**:1500-10.

8 Guo Y, Apostolakis S, Blann AD, et al. Validation of contemporary stroke and bleeding risk stratification scores in non-anticoagulated Chinese patients with atrial fibrillation. *International journal of cardiology* 2013;**168**:904-9.

9 Lip GY, Nieuwlaat R, Pisters R, et al. Refining clinical risk stratification for predicting stroke and thromboembolism in atrial fibrillation using a novel risk factor-based approach: the euro heart survey on atrial fibrillation. *Chest* 2010;**137**:263-72.

10 Friberg L, Skeppholm M, Terent A. Benefit of anticoagulation unlikely in patients with atrial fibrillation and a CHA2DS2-VASc score of 1. *Journal of the American College of Cardiology* 2015;**65**:225-32.

11 Forslund T, Wettermark B, Wandell P, et al. Risks for stroke and bleeding with warfarin or aspirin treatment in patients with atrial fibrillation at different CHA(2)DS(2)VASc scores: experience from the Stockholm region. *European journal of clinical pharmacology* 2014;**70**:1477-85.

12 Connolly SJ, Ezekowitz MD, Yusuf S, et al. Dabigatran versus warfarin in patients with atrial fibrillation. *The New England journal of medicine* 2009;**361**:1139-51.

13 Patel MR, Mahaffey KW, Garg J, et al. Rivaroxaban versus warfarin in nonvalvular atrial fibrillation. *The New England journal of medicine* 2011;**365**:883-91.

14 Granger CB, Alexander JH, McMurray JJ, et al. Apixaban versus warfarin in patients with atrial fibrillation. *The New England journal of medicine* 2011;**365**:981-92.

15 Giugliano RP, Ruff CT, Braunwald E, et al. Edoxaban versus warfarin in patients with atrial fibrillation. *The New England journal of medicine* 2013;**369**:2093-104.
